# Hepatic injury and hepatic failure adverse events in 3,4-methylenedioxymethamphetamine users reported to the FDA Adverse Event Reporting System

**DOI:** 10.3389/fpsyt.2024.1414622

**Published:** 2024-06-18

**Authors:** Tigran Makunts, Ruben Abagyan

**Affiliations:** Skaggs School of Pharmacy and Pharmaceutical Sciences, University of California, San Diego, La Jolla, CA, United States

**Keywords:** DILI (drug-induced liver injury), MDMA, DDI (drug-drug interaction), FAERS, adverse events, MDMA (3,4-methylenedioxymethamphetamine)

## Abstract

3,4-Methylenedioxymethamphetamine (MDMA) is being investigated in controlled clinical trials for use as an adjunct medication treatment for post-traumatic stress disorder. MDMA is metabolized by N-demethylation, primarily by CYP2D6, to its main inactive metabolite, 4-hydroxy-3-methoxymethamphetamine. It is also metabolized to a lesser extent by CYP1A2, CYP2B6, and CYP3A4 to its active metabolite, 3,4-methylenedioxyamphetamine. Considering the extensive hepatic metabolism and excretion, MDMA use in psychiatry raises concerns over drug-induced liver injury (DILI), a rare but dangerous event. Majority of the drugs withdrawn from the market for liver injury caused death or transplantation at frequencies under 0.01%. Unfortunately, markers for liver injury were not measured in most published clinical trials. At the same time, no visible DILI-related symptoms and adverse events were observed. Idiosyncratic DILI cases are rarely registered during clinical trials due to their rare nature. In this study, we surveyed a larger, over 1,500, and a more diverse set of reports from the FDA Adverse Event Reporting System and found 23 cases of hepatic injury and hepatic failure, in which MDMA was reported to be taken in addition to one or more substances. Interestingly, 22 out of 23 cases had one or more listed drugs with a known DILI concern based on the FDA’s DILIrank dataset. Furthermore, only one report had MDMA listed as the primary suspect. Considering the nearly 20 million doses of MDMA used annually, this single report is insufficient for establishing a significant association with DILI.

## Introduction

MDMA or 3,4-methylenedioxymethamphetamine is currently a controlled Class A substance in the United Kingdom and Schedule I substance in the United States and the European Union. Based on efficacy and safety findings in multiple clinical trials (1–5), there is an ever-increasing interest in MDMA use in psychiatry.

MDMA is metabolized through N-demethylation primarily by cytochrome P450 2D6 (CYP2D6). This pathway produces its main inactive metabolite 4-hydroxy-3-methoxymethamphetamine. MDMA is also metabolized to a lesser extent by CYP1A2, CYP2B6, and CYP3A4 to its active metabolite, 3,4-methylenedioxyamphetamine. Most of the drugs associated with drug-induced liver injury (DILI) are metabolized through the hepatic pathway (6). However, there is no correlation with any specific CYP450 metabolic pathway and liver injury or failure. Most DILI cases are of idiosyncratic nature (7, 8) with a few exceptions such as in the case with acetaminophen (paracetamol) (9). Although DILI has not been observed in controlled clinical trials using MDMA (1, 2, 10, 11), considering the rare nature of this adverse event [13.9–24.0 per 100,000, and severe cases under 10 per 100,000 (12, 13)], it is rarely captured in clinical trials and cannot be completely ruled out. There have been published case reports of liver injury and liver failure with detectable MDMA levels, suggesting a possible association of its use with DILI (14–20). However, in every single case, the confounding factors, such as known DILI-concern concomitant drugs and infectious or other hepatitis, had not been addressed to justify causality assessment of MDMA as a culprit.

The lack of any concrete evidence of MDMA association with DILI warranted a further evaluation of MDMA user reports associated with liver injury and liver failure from the United States Food and Drug Administration Adverse Event Reporting System (FDA AERS or FAERS) reported to the FDA through MedWatch (21). In this study, FAERS reports were evaluated for the presence of MDMA as the sole reported drug and for the presence of any additional drugs with a known association with DILI based on the DILIrank database (13, 22).

## Methods

### FDA Adverse Event Reporting System

The FDA Adverse Event Reporting System (FAERS) is dataset repository, which hosts adverse events (AEs) submitted to the FDA through MedWatch AE forms 3,500 and 3500A by manufacturers, consumers, lawyers, and healthcare professionals (21).

Although initially intended for postmarketing safety surveillance of approved drugs and biologics, FAERS is a useful and significant source of safety information on drugs still under investigation, drugs such as Schedule I/Class A substances not yet approved by regulatory authorities, but available to the public through illicit means.

### Case selection

FAERS/AERS quarterly datasets, each including separate tables for demographics, country, drug, indication, outcome, reaction, and report source, were downloaded individually from the FDA public repository in dollar sign-separated text format (23–25). Unix shell scripts were used for data restructuring and filtering (26).

At the time of the analysis, FAERS/AERS contained 19,190,582 reports from January 2004 to March 2023. A total of 1,575 reports involving reported MDMA/ecstasy were selected for further analysis. Liver failure and liver injury FDA medical queries (FMQs) were used to filter out the cases of interest (see **Table 1** for a comprehensive list of the search terms). Cases with any of the FMQs reported to be associated with viral or ischemic hepatitis were excluded. Each individual remaining case was reviewed for exclusion of any duplicates by multiple reporters.

**Table 1:** FAERS database search (June 2023) keywords and search terms.

MDMA and Metabolite Search Terms
Chemical names: MDMA, 3,4-MDMA, midomaphetamine, midomafetamine, methylenedioxymethamphetamine, 3,4-methylenedioxymethamphetamine, 3,4methylenedioxynmethylamphetamine Common or street names (may include if MDMA presence confirmation described in studies): Adam, Beans, Clarity, Disco Biscuit, E, Ecstasy, Eve, Go, Hug Drug, Lover’s Speed, Molly, Peace, STP, X, and XTC Metabolites: MDA, 3,4methylenedioxyamphetamine, HMMA, 4Hydroxy3methoxymethamphetamine, HMA, 4hydroxy3methoxyamphetamine, HHA, DHA, 3,4dihydroxyamphetamine, αMeDA, alphamethyldopamine.
Hepatic Injury and Hepatic Failure FMQs (narrow) PTs
Hepatic injury narrow-FMQ scope: Acquired hepatocerebral degeneration, acute hepatic failure, acute yellow liver atrophy, alanine aminotransferase increased, allergic hepatitis, alloimmune hepatitis, ammonia increased, aspartate aminotransferase increased, biliary ascites, biliary cirrhosis, biliary cirrhosis primary, biliary fibrosis, bilirubin conjugated increased, Child–Pugh–Turcotte score abnormal, Child–Pugh–Turcotte score increased, chronic hepatic failure, chronic hepatitis, coma hepatic, cryptogenic cirrhosis, cytolytic hepatitis, drug-induced liver injury, fetor hepaticus, gallbladder varices, gastric variceal injection, gastric variceal ligation, gastric varices, gastric varices hemorrhage, granulomatous liver disease, hepatic cirrhosis, hepatic encephalopathy, hepatic failure, hepatic fibrosis marker abnormal, hepatic function abnormal, hepatic hydrothorax, hepatic hypertrophy, hepatic infiltration eosinophilic, hepatic lymphocytic infiltration, hepatic necrosis, hepatic pain, hepatic steato-fibrosis, hepatic vascular resistance increased, hepatitis, hepatitis acute, hepatitis cholestatic, hepatitis chronic active, hepatitis chronic persistent, hepatitis fulminant, hepatitis mumps, hepatitis toxic, hepatobiliary disease, hepatobiliary scan abnormal, hepatocellular damage, hepatocellular injury, hepatomegaly, hepatopulmonary syndrome, hepatorenal failure, hepatorenal syndrome, hepatosplenomegaly, hepatotoxicity, hyperammonemia, hyperammonemic crisis, intestinal varices, intestinal varices hemorrhage, intrahepatic portal hepatic venous fistula, jaundice hepatocellular, Kayser–Fleischer ring, liver disorder, liver injury, minimal hepatic encephalopathy, mixed hepatocellular-cholestatic injury, mixed liver injury, non-alcoholic fatty liver, nonalcoholic fatty liver disease, non-alcoholic steatohepatitis, non-cirrhotic portal hypertension, edema due to hepatic disease, esophageal varices hemorrhage, peripancreatic varices, periportal edema, portal fibrosis, portal hypertension, portal hypertensive colopathy, portal hypertensive enteropathy, portal hypertensive gastropathy, portal shunt, portal triaditis, portal vein cavernous transformation, portal vein dilatation, portal vein flow decreased, portal vein pressure increased, portopulmonary hypertension, primary biliary cholangitis, regenerative siderotic hepatic nodule, Reye’s syndrome, splenic varices, splenic varices hemorrhage, steatohepatitis, stomal varices, subacute hepatic failure, total bile acids increased, ultrasound liver abnormal, varices esophageal, varicose veins of abdominal wall, white nipple sign, X-ray hepatobiliary abnormal Hepatic failure narrow-FMQ Narrow scope: Acute hepatic failure, acute on chronic liver failure, acute yellow liver atrophy, ammonia increased, chronic hepatic failure, coma hepatic, hepatic encephalopathy, hepatic failure, hepatitis fulminant, hepatopulmonary syndrome, hepatorenal failure, hepatorenal syndrome, hyperammonemic crisis, liver dialysis, minimal hepatic encephalopathy, subacute hepatic failure

## Results

There was a total of seven unique liver failure narrow-FMQ AE cases in the FEARS database. All of these cases were reported by healthcare professionals, and one of the seven was also reported by a consumer. Four out of seven cases were overdose cases, and six of these reported one or more drugs known to be associated with liver injury according to the DILIrank dataset. No cases reported MDMA as a “primary suspect” of the AEs. One overdose case had cocaine, and MDMA both reported as “secondary suspects” with no “primary suspect” listed (**Table 2**).

**Table 2:** Hepatic failure FDA Medical Query cases.

Cases reported by healthcare professionals									
CaseID # (number of submission)	Age (yrs.)	Weight (kg)	Sex Male (M) Female (F)	Country	Concomitant medications/drugs	Indication	Adverse events	Outcome code	Reporter (occupation code)
1 (6)	16	unk	M	US	ps: acetaminophen and hydrocodone bitartrate ss: amphetamine ss: cocaine ss: marijuana ss: methamphetamine hcl ss: methylenedioxymethamphetamine	unk	disseminated intravascular coagulation, **hepatorenal failure**, hyperthermia malignant, hypoglycemia, hypoxic ischemic encephalopathy, intentional drug misuse, multi organ failure, multiple drug overdose intentional, rhabdomyolysis, serotonin syndrome, shock, death	DE, HO, RI	LT, HP, MD
2	16	59	F	CA	ps: Abilify (aripiprazole) ss: methylenedioxymethamphetamine c: desogestrel	unk	cholestasis, **hepatitis fulminant**, intensive care, liver transplant	HO, OT, LT	PH
3(11)	21	56	M	DE	ps: pregabalin, ps: clonazepam** ss: methylenedioxymethamphetamine	drug abuse, unk	blood lactic acid increased, coagulopathy, dehydration, drug abuse, gastrointestinal hemorrhage, generalized tonic clonic seizure, hemoglobin decreased, hemorrhage, **hepatic failure**, hyperpyrexia, hyperthermia, hypoglycemia, mydriasis, nervous system disorder, platelet count decreased, renal failure, rhabdomyolysis, tachycardia, toxicity to various agents acute kidney injury, muscle hemorrhage, tonic clonic movements	HO, LT, OT	PH, HP
4	32	unk	M	IT	ps: acetaminophen ss: citalopram ss: midomafetamine ss: trazodone	unk	**acute hepatic failure**, drug abuse, drug interaction, **hepatotoxicity**, overdose, toxicity to various agents, death	DE, OT	HP
5	49	unk	M	FR	ps: acetaminophen ss: cocaine ss: midomafetamine	unk	acute kidney injury, drug abuse, **hepatitis fulminant,** hypoxia, multiple organ dysfunction syndrome, septic shock, death	DE, OT	MD
6	unk	unk	unk	GB	ss: cocaine ss: ecstasy	unk	**acute hepatic failure,** overdose, toxicity to various agents	HO, LT	OT
7	21	unk	M	US	ps: propofol: IV drip, ss: methylenedioxymethamphetamine: PO c:n acetylcysteine	unk	**acute hepatic failure,** cerebral hemorrhage, encephalopathy, hyperhidrosis, muscle rigidity, renal failure, rhabdomyolysis	HO	HP, LT. OT
Cases reported by consumers									
1***	16	60	F	CA	ps: Abilify (aripiprazole):PO ss: methylenedioxymethamphetamine c: desogestrel: PO c: ethinylestradiol	unk	cholestasis, **hepatitis fulminant,** intensive care	HO, LT, OT	CN

**Variable primary suspect assignments by multiple reporters. Unk, unknown; M, male; F, female; IE, Ireland; US, United States; AT, Austria; HK, Hong Kong; FR, France; CA, Canada; DE, Germany; IT, Italy; ES, Spain; PS, primary suspect; GB, Great Britain; SS, secondary suspect; C, concomitant; I, interacting; NAFLD, nonalcoholic fatty liver disease; HO, hospitalization; LT (outcome section), life threatening; DE, death; RI, requiring intervention; OT, other medically important event; MD, physician; HP (or OT), other healthcare professional; PH, pharmacist; LT (reporter section); literature. ***Case reported both by healthcare professional and consumer. Specific hepatic failure narrow-FMQs terms are shown in bold.

There were 16 unique liver injury narrow-FMQ AE cases in the FEARS database, including 14 cases reported by healthcare professionals, one by a lawyer, and one by a consumer. Five out of 16 cases were overdose cases, and as many as 15 out of the 16 cases had reported one or more drugs known to be associated with liver injury according to the DILIrank. One case was reported with MDMA as a “primary suspect” with alcohol listed as a “secondary suspect” (**Table 3**). The cases for both hepatic failure and injury are summarized in **Table 4**.

**Table 3:** Hepatic injury FDA Medical Query cases reported by healthcare professionals.

Cases reported by healthcare professionals									
Case # (number of duplicates)	Age (Yrs.)	Weight (kg)	Sex Male (M) Female (F)	Country	Concomitant medications/drugs	Indication	Adverse events	Outcome code	Reporter (occupation code)
1	unk	63.5	M	IE	ps: Invega (paliperidone) ss: alcohol ss: ecstasy ss: olanzapine	schizophrenia	adverse reaction, agitation, anaphylactic reaction, **aspartate aminotransferase increased,** blood creatine phosphokinase increased, blood lactate dehydrogenase increased, chest pain, multiple drug overdose, psychotic disorder, treatment noncompliance, white blood cell count increased	HO, LT	MD
2	20	65	F	AT	ps: Seroquel: (quetiapine) ss: Depakine (valproic acid) c: alcohol c: cocaine c: dhc c: ecstasy c: speed	unk	**ammonia increased,** drug abuse, hypotension, sinus bradycardia, somnolence	HO	MD
3	21	74.8	M	US	ps: mdma ss: alcohol	unk	agitation, confusional state, convulsion, **hepatotoxicity**, hyperthermia, international normalized ratio increased, **liver injury,** rhabdomyolysis	HO	PH
4	unk	unk	unk	HK	ps: Ketalar (ketamine) ss: alcohol ss: cocaine ss: crystal methamphetamine ss: ecstasy	drug diversion	biliary dilatation, biliary tract disorder, drug abuse, **liver injury, ** **portal fibrosis**, urinary tract disorder	OT	MD
5	24	unk	M	FR	ps: Zelboraf (vemurafenib) ss: Cotellic: (cobimetinib) c: alprazolam c: ecstasy	malignant melanoma	diarrhea, **hepatitis cholestatic**, maculopathy, renal failure	OT	MD
6(2)	55	unk	F	IT	ps: acetaminophen ps: bromazepam** ss: carbamazepine ss: trazodone c:methylenedioxymethamphetamine c:morphine	Headache, unk	atrial fibrillation, bradycardia, bradypnea, **hepatitis acute**, hypokalemia, hypotension, hypothermia, loss of consciousness, mydriasis, overdose, product use in unapproved indication	HO, LT, OT	HP
7(7)	27	unk	M	FR	ps: quetiapine, ps: buprenorphine** ss: crack cocaine ps: Lyrica (pregabalin)** ss: mdma	unk	bradypnea, coma, **hepatic cytolysis (cytolytic hepatitis)**, rhabdomyolysis	HO	MD
8	unk	unk	unk	AU	ps: fentanyl ss: alcohol ss: buprenorphine ss: cannabinol ss: cocaine ss: codeine ss: hydromorphone ss: methadone ss: methamphetamine ss: midomafetamine ss: morphine ss: olanzapine ss: oxycodone ss: promethazine ss: quetiapine ss: tapentadol ss: tramadol hcl	unk	arteriosclerosis coronary artery, aspiration, asthma, cardiac valve disease, cardiomegaly, cardiomyopathy, emphysema, fibrosis, **hepatic cirrhosis,** **hepatic fibrosis,** **hepatic hypertrophy,** **hepatic steatosis (NAFLD),** **hepatitis,** intentional self-injury, kidney fibrosis, nephrosclerosis, overdose, pneumonia, pulmonary edema, toxicity to various agents, ventricular hypertrophy, death	DE, OT	HP
9(3)	25	unk	M	US	ps: sertraline ss: cocaine ss: midomafetamine c: St John’s wort	unk	abdominal pain upper, aggression, **alanine aminotransferase increased,** **aspartate aminotransferase increased,** blood potassium decreased, decreased appetite, disorientation, drug abuse, electrocardiogram qt prolonged, nausea, oxygen saturation decreased, serotonin syndrome, vomiting, weight decreased	OT	HP, LT, OT
10(4)	34	unk	M	AU	ps: citalopram ss: 6-acetylmorphine (heroine) ss: alcohol ss: midomafetamine	unk	**hepatic steatosis (NAFLD)**, prostatitis, serotonin syndrome, toxicity to various agents, death	DE, OT	HP, OT
11(3)	26	unk	M	UK	ps: codeine ss: acetaminophen: PO c: benzodiazepine c: ecstasy	unk	coagulopathy, **hepatic function abnormal,** **liver disorder,** overdose	HO	OT, LT
12	23	unk	M	ES	ps: ritonavir c: atazanavir c: Truvada (emtricitabine + tenofovir) i: 3,4-methylenedioxymethamphetamine	unk	coma scale abnormal, convulsion, depressed level of consciousness, disseminated intravascular coagulation, dizziness, drug interaction, drug level increased, **hepatic function abnormal,** hyperthermia, malaise, renal failure, rhabdomyolysis, toxicity to various agents, vision blurred, vomiting	HO, LT, OT	OT, LT, HP
13	21	unk	F	GB	ps: paracetamol ss: alcohol ss: methylenedioxymethamphetamine	unk	**hepatic function abnormal**, overdose, toxicity to various agents	HO	HO, LT,OT
14	28	unk	M	GB	ps: Ketalar(ketamine) ss: ecstasy c: alimemazine c: amphetamine c: trazodone: PO	drug abuse Insomnia depression	bladder wall calcification, **liver function test abnormal,** pelvic pain, pollakiuria	OT	OT
Cases reported by lawyers and consumers									
1	15	100	M	US	ps: Accutane (isotretinoin):PO c: cocaine c: ecstasy c: marijuana c: methamphetamine hcl c: prednisone tab c: Zithromax (azithromycin)	Acne	abnormal behavior, affect lability, aggression, **alanine aminotransferase increased,** alcoholism, anger, anhedonia, anxiety, automatism, bone disorder, bronchitis, delirium, delusion, depersonalization, depression, distractibility, disturbance in attention, drug abuser, failed examinations, fatigue, fear, feeling guilty, feeling of despair, feelings of worthlessness, flight of ideas, gastrointestinal disorder, headache, high density lipoprotein decreased, hypertriglyceridemia, impulse control disorder, inflammatory bowel disease, injury, insomnia, irritability, irritable bowel syndrome, laryngotracheo bronchitis legal problem major depression, mental disorder, muscle injury, performance fear, pharyngitis, respiratory disorder, restlessness, rhinorrhea, self-esteem decreased, sleep disorder, social problem, theft, tinea versicolor, truancy, xerosis	DS	LW
2	19	unk	M	CA	ps: Dilaudid: IV(hydromorphone) ss: cocaine ss: marijuana ss: methylenedioxymethamphetamine ss: morphine sulfate ss: Oxycontin(oxycodone) ss: Percocet(oxycodone/acetaminophen) ss: Ritalin(methylphenidate) ss: sleeping pills	unk	drug dependence, euphoric mood, infection, **jaundice,** **liver injury,** mobility decreased, edema peripheral, overdose, substance abuse	HO, OT	CN

**Variable primary suspect assignments by multiple reporters. Unk, unknown; M, male; F, female; IE, Ireland; US, United States; AT, Austria; HK, Hong Kong; FR, France; CA, Canada; DE, Germany; IT, Italy; ES, Spain; PS, primary suspect; GB, Great Britain; SS, secondary suspect; C, concomitant; I, interacting; NAFLD, nonalcoholic fatty liver disease; HO, hospitalization; LT (outcome section), life threatening; DE, death; RI, requiring intervention; OT, other medically important event; MD, physician; HP (or OT), other healthcare professional; PH, pharmacist; LT (reporter section), literature; CN, consumer; LW, lawyer. Specific liver injury narrow-FMQ terms are shown in bold.

**Table 4:** Summary of FAERS hepatic failure and hepatic injury FMQ reports.

	Reported by healthcare professionals	Reported by consumers and lawyers	Total number of cases
Hepatic failure cases	7	1***	7
Hepatic injury cases	14	2	16
Cases with ecstasy as sole reported drug	0	0	0
Polydrug use/polypharmacy cases	21	3***	23
Cases with reported drug(s) listed in FDA DILIrank dataset as DILI concern drugs	hepatic injury cases—13 hepatic failure cases—6	hepatic injury cases—2 hepatic failure cases—1***	hepatic injury cases—15 hepatic failure cases—6
Ecstasy/MDMA reported primary suspect cases	1 (alcohol as only secondary suspect)	0	1
Ecstasy/MDMA as a secondary suspect drug	hepatic injury cases—8 hepatic failure cases—7	hepatic injury cases—1 hepatic failure cases—1***	hepatic injury cases—9 hepatic failure cases—7
Ecstasy/MDMA as a concomitant drug	hepatic injury cases—4 hepatic failure cases—0	hepatic injury cases—1 hepatic failure cases—0	hepatic injury cases—5 hepatic failure cases—0
Ecstasy/MDMA as an interacting drug	hepatic injury cases—1 hepatic failure cases—0	hepatic injury cases—0 hepatic failure cases—0	hepatic injury cases—1 hepatic failure cases—0
Overdose cases	8	1	9

***Duplicate report by pharmacists and consumer not included in the total count.

## Discussion

In this study, we evaluated liver failure and liver injury cases reported as associated with a list of drugs including MDMA, from the United States Food and Drug Administration FAERS. We found no cases where MDMA was the sole reported compound. This was in line with the absence of liver injury or liver failure AEs in the clinical trials. Additionally, we observed a limited number of 23 (seven hepatic failure and 16 hepatic injury cases) originating from a list of drugs including MDMA in the last ~18 years. Of these 23 cases, 21 listed one or more co-reported drugs associated with liver injury or liver failure according to DILIrank (13) (**Table 5**). MDMA was listed as a primary suspect only in a single case, which contained alcohol use, a known substance to cause liver damage, concurrent with MDMA. There is still the possibility that MDMA may have played a role in the other drugs’ hepatotoxic effects due to a potential CYP2D6-mediated drug–drug interaction (27, 28). However, considering the worldwide widespread MDMA use (~20 million annually) (29), the single report with liver injury/failure reported to the FAERS database was insufficient to establish a meaningful association signal.

**Table 5:** Drugs of concern according to DILIrank list in the FAERS liver injury and liver failure FMQ reports.

Drug	Label section	FDA DILI concern
Acetaminophen	Warnings and precautions	Most DILI concern
Amphetamine	No match	Less DILI concern
Alprazolam	Adverse reactions	Less DILI concern
Aripiprazole	Adverse reactions	Ambiguous DILI concern
Atazanavir	Warnings and precautions	Less DILI concern
Azithromycin	Adverse reactions	Less DILI concern
Bromazepam	Unlisted	
Buprenorphine	Unlisted	
Carbamazepine	Warnings and precautions	Most DILI concern
Citalopram	Adverse reactions	Less DILI concern
Clonazepam	Adverse reactions	Less DILI concern
Emtricitabine	Warnings and precautions	Ambiguous DILI concern
Fentanyl	Unlisted	
Hydromorphone	Adverse reactions	Ambiguous DILI concern
Isotretinoin	Warnings and precautions	Most DILI concern
Ketamine	N/A	Less DILI concern
Methadone	Adverse reactions	Ambiguous DILI concern
Methylphenidate	Adverse reactions	Less DILI concern
Olanzapine	Adverse reactions	Less DILI concern
Prednisone	Adverse reactions	Less DILI concern
Pregabalin	N/A	Less DILI concern
Promethazine	Adverse reactions	Less DILI concern
Propofol	Adverse reactions	Less DILI concern
Quetiapine	Warnings and precautions	Less DILI concern
Ritonavir	Warnings and precautions	Most DILI concern
Sertraline	Adverse reactions	Less DILI concern
Tenofovir	Warnings and precautions	Less DILI concern
Tramadol	Adverse reactions	Ambiguous DILI concern
Trazodone	Adverse reactions	Less DILI concern
Valproic acid	Boxes warning	Most DILI concern
Vemurafenib	Unlisted	

Drug-Induced Liver Injury Rank (DILIrank) Dataset: https://www.fda.gov/science-research/liver-toxicity-knowledge-base-ltkb/drug-induced-liver-injury-rank-dilirank-dataset.

## Study limitations

Reporting to FAERS is mostly voluntary, apart from spontaneous reports forwarded from the manufacturers/authorization holders. Thus, the dataset represents only a subset of actual cases and should not be confused with absolute population frequencies. Most of the cases are not clinically assessed for causality. There was no consistent means for reporters to provide information on drug identification or detection. Since manufacture and distribution of MDMA is not regulated, it is uncertain whether the chemical compound listed in the cases could be confirmed as MDMA or MDMA laced with another compound.

## Conclusion

In summary, reported use of MDMA as the only administered drug produced a single report of liver injury or liver failure in the FAERS system; it was far more common for hepatotoxicity-related AEs to arise when MDMA was reportedly combined with an additional substance with well-documented DILI-concern (22). The current findings in the FAERS system are in line with the failure of clinical trials to report DILI.

## Data availability statement

Publicly available datasets were analyzed in this study. This data can be found here: https://fis.fda.gov/extensions/FPD-QDE-FAERS/FPD-QDE-FAERS.html.

## Ethics statement

Ethical approval was not required for the study involving humans in accordance with the local legislation and institutional requirements. Written informed consent to participate in this study was not required from the participants or the participants’ legal guardians/next of kin in accordance with the national legislation and the institutional requirements.

## Author contributions

TM: Writing – review & editing, Writing – original draft, Validation, Methodology, Investigation, Formal analysis, Conceptualization. RA: Writing – review & editing, Writing – original draft, Supervision, Software, Resources, Project administration, Methodology, Investigation, Funding acquisition, Data curation, Conceptualization.
